# Neural efficiency as a function of task demands^☆^

**DOI:** 10.1016/j.intell.2013.09.005

**Published:** 2014-01

**Authors:** Beate Dunst, Mathias Benedek, Emanuel Jauk, Sabine Bergner, Karl Koschutnig, Markus Sommer, Anja Ischebeck, Birgit Spinath, Martin Arendasy, Markus Bühner, Heribert Freudenthaler, Aljoscha C. Neubauer

**Affiliations:** a Department of Psychology, University of Graz, Austria; b Department of Leadership and Entrepreneurship, University of Graz, Austria; c Department of Psychology, Ludwig-Maximilians-University Munich, Germany; d Department of Psychology, Ruprecht-Karls-University Heidelberg, Germany

**Keywords:** fMRI, Intelligence, Neural efficiency, Tailored testing, Task difficulty

## Abstract

The neural efficiency hypothesis describes the phenomenon that brighter individuals show lower brain activation than less bright individuals when working on the same cognitive tasks. The present study investigated whether the brain activation–intelligence relationship still applies when more versus less intelligent individuals perform tasks with a comparable person-specific task difficulty. In an fMRI-study, 58 persons with lower (*n* = 28) or respectively higher (*n* = 30) intelligence worked on simple and difficult inductive reasoning tasks having the same person-specific task difficulty. Consequently, less bright individuals received sample-based easy and medium tasks, whereas bright subjects received sample-based medium and difficult tasks. This design also allowed a comparison of lower versus higher intelligent individuals when working on the same tasks (i.e. sample-based medium task difficulty). In line with expectations, differences in task performance and in brain activation were only found for the subset of tasks with the same sample-based task difficulty, but not when comparing tasks with the same person-specific task difficulty. These results suggest that neural efficiency reflects an (ability-dependent) adaption of brain activation to the respective task demands.

## Highlights

• The effect of task difficulty on neural efficiency phenomenon was studied.
• Tasks of same sample-based and same person-specific difficulty were contrasted.
• Performance differences were only found for tasks with same sample-based difficulty.
• Activation differences were only found for tasks with same sample-based difficulty.
• Neural efficiency may reflect ability-dependent adaption of brain.

## 1 Introduction

The study of human intelligence is an old research tradition, starting over a century ago. Spearman (1904) proposed that a general ability factor (g) determines performance in a variety of cognitive activities, including perception, attention, memory, language and thought. The best indicator of general ability in Spearman's sense is fluid intelligence (Carroll, 1993, Cattell, 1971). Fluid intelligence (gf) tests measure the ability to solve novel problems that depend relatively little on stored knowledge or the ability to learn. In order to correctly answer fluid intelligence tasks it is necessary to actively maintain domain-specific information and domain-general attention or “executive” control of ongoing processes. Consequently, it can be assumed that gf is related to attentional control and working memory (Conway et al., 2002, Kane and Engle, 2003). Among different tests the Raven's Progressive Matrices are often considered the best available measure of gf. Moreover, Arendasy, Hornke, et al. (2008) found the numerical inductive reasoning task to be a good indicator of fluid intelligence.

The focus of the current study is on the neurobiological underpinnings of fluid intelligence. Numerous structural and functional brain imaging studies have been conducted so far to investigate neurobiological correlates of intelligence. A review of mostly structural findings led to the formulation of the parieto-frontal integration theory of intelligence (P-FIT; Jung & Haier, 2007). In the attempt to locate intelligence in the human brain Jung and Haier (2007) reviewed 37 neuroimaging studies of intelligence. They found that mostly frontal and parietal areas of the cortex are related to intelligence. After a first sensory analysis of incoming information in the occipital cortex the sensory information will be abstracted and elaborated in parietal areas. Testing hypotheses concerning a problem and finding the best solution are conducted in the frontal cortex, which interacts with the parietal cortex. The anterior cingulate cortex accounts for the response selection and inhibits competing ones. Moreover, the efficiency of these cognitive processes may depend on the integrity of white matter connections between the regions (Jung & Haier, 2007). Barbey, Colom, Paul, and Grafman (2013) demonstrated that damage within white matter fiber tracts (superior longitudinal/arcuate fasciculus connecting frontal and parietal cortices) impairs fluid intelligence. To sum up, the fronto-parietal integration theory provides an important framework for the study of human intelligence, since its formulation has been supported by numerous studies (e.g., Colom et al., 2009; for a review see, Deary, Penke, & Johnson, 2010).

Besides the P-FIT (Jung & Haier, 2007), which is based mostly on evidence of structural correlates of intelligence, the neural efficiency hypothesis provides another influential account to explain the relationship of intelligence and brain activation. The neural efficiency hypothesis has first been stated by Haier et al. (1988) who observed that participants' intelligence scores and regional metabolic rates are negatively correlated between − .48 and − .84 (for different brain areas), indicating that participants with higher cognitive ability display a lower energy consumption of the brain. Based on a later confirmation of this finding, Haier introduced the neural efficiency hypothesis that claims that brighter individuals show more efficient brain functioning than less intelligent individuals (Haier, Siegel, Tang, Abel, & Buchsbaum, 1992). In contrast to structural correlates of intelligence, evidence for functional correlates of intelligence is contradictory. Neubauer and Fink (2009) provided a thorough review of the pertinent research showing that out of a total of 54 studies (employing diverse methods of functional brain imaging like fMRI, PET, EEG and a variety of cognitive tasks) the majority (k = 29) confirmed the neural efficiency hypothesis (i.e. showed negative correlations between brain activation and intelligence).

However, as shown by Neubauer and Fink (2009), another group of studies (k = 16) produced mixed evidence, i.e. found the expected inverse brain activation–intelligence relationship only when moderating variables were considered, while another smaller group of studies (k = 9) disconfirmed the hypothesis by finding positive relationships between intelligence and brain activation. Thus, there is evidence pro and contrary the neural efficiency hypothesis. Moderator variables like task complexity (Doppelmayr et al., 2005), level of expertise in the task (state of learning) (Grabner, Neubauer, & Stern, 2006), sex (Dunst et al., 2013, Neubauer et al., 2002, Neubauer et al., 2005), and brain area (Jaušovec & Jaušovec, 2004) were proposed to explain the conflicting evidence.

One of the most prominent variables moderating the neural efficiency effect is task difficulty. Doppelmayr et al. (2005) confirmed the expected inverse IQ–brain activation relationship for easy tasks, whereas a tendency in the opposite direction was observed for the more difficult items. Such an interaction between task difficulty and intelligence was also found by Lipp et al. (2012) and Preusse, Van der Meer, Deshpande, Krueger, and Wartenburger (2011). With increasing task difficulty stronger activation for participants with high intelligence was found. This interaction was located in an occipital-temporal region (Preusse et al., 2011) and in right frontal and right inferior parietal regions (Lipp et al., 2012). Similar results were found by Rypma, Berger, and D'Esposito (2002). While high-performing participants showed activation increases with increasing difficulty in the lateral PFC, low-performing participants showed higher activation but minimal difficulty-dependent increase. These findings suggest that cortical activation is modulated by effort requirements. A common explanation for this phenomenon is that brighter individuals are prepared to invest more effort when working on difficult items than individuals with lower ability. This might be due to the circumstance that individuals with lower ability anticipate their task-related disability and in consequence invest less effort to solve the task. Larson, Haier, Lacasse, and Hazen (1995) were the first to test whether the perceived level of task difficulty is responsible for individual differences in brain activation. In a PET study, participants' task was to solve Backwards Digit Span tasks that were tailored to the participants' ability level in order to eliminate group differences in effort requirements. Results demonstrated that brighter individuals increased their metabolic rates during the difficult task whereas less intelligent individuals decreased their metabolic rates.

Taken together, there is no unequivocal evidence that higher intelligence is generally related to lower brain activation as assumed by the neural efficiency hypothesis. Prior evidence supports the notion that task difficulty moderates the relation between intelligence and brain activation. In line with that research, we tested whether neural efficiency is due to differences in intelligence per se, or rather due to differences in task demands such as person-specific task difficulty. In other words the question is: Does neural efficiency hold true when people work on tasks with the same person-specific task difficulty?

Task difficulty is known to be related to response time as well as effort investment, two aspects that may also account for differences in brain activation. Based on Brehm's theory (Brehm & Self, 1989) effort expenditure directly depends on task difficulty and motivation. The more difficult the task, the more effort people invest in the task. However, effort investment is only beneficial up to a certain point (of moderate intensity of motivation), above that point performance should again decline (following Yerkes & Dodsons's inverted U-shaped function; Yerkes & Dodson, 1908). Neurocognitive research on individual effort showed that increased mental effort is frequently associated with increased brain activation, particularly in frontal regions (Gevins & Smith, 2000).

### 1.1 Aim of this study

In studies confirming the neural efficiency hypothesis, typically all participants completed the same cognitive tasks. In light of individual differences regarding intelligence, the same task is easier for individuals with higher cognitive ability, whereas it is more difficult for less intelligent individuals. In other words, although all individuals work on tasks with the same sample-based difficulty, the person-specific level of task difficulty differs depending on the individual's level of intellectual ability. Since the person-specific level of task difficulty can be expected to be associated with the time and effort spent on the cognitive task, it could also be responsible for observed differences in brain activation.

The current study aims at testing whether the person-specific level of task difficulty accounts for individual differences in neural efficiency. Therefore, brain activation is investigated using tasks with a relatively easy difficulty level versus tasks with a relatively difficult level of task difficulty. Consequently, in the present study a person-specific rather than a sample-based approach was used for assigning the task difficulty. According to the person-specific approach the task difficulty is defined relative to an individual's intellectual ability. Using this approach ensures that the perceived level of task difficulty is the same for individuals with varying intellectual ability. As a consequence, brighter individuals compared to less intelligent ones have to work on tasks which are actually more difficult (i.e., with respect to sample-based task difficulty). Additionally, tasks and participants were selected in a way that the easy items for more intelligent individuals were the same items as the difficult items for less intelligent individuals. Consequently, the typical comparison between brighter and less bright individuals working on the same tasks was still possible (sample-based approach).

The task employed in the current study is the number series completion task which is used to assess numerical inductive reasoning. The number series used in this study consists of automatically generated, Rasch-calibrated items which provide the opportunity to estimate the task difficulty relative to different levels of intelligence (Rasch, 1980).

The main aim of the study was to refine the neural efficiency hypothesis. The crucial research question was whether neural efficiency is not only a function of differences in intelligence but also a function of task demands such as person-specific task difficulty. Neural efficiency during number processing was investigated under two experimental conditions working on sample-based same difficult items or working on person-specific same difficult items. We expected to find no differences in brain activation between intelligence groups working on tasks with same person-specific task difficulty. When working on tasks with same person-specific task difficulty, we expect that brighter individuals show lower brain activation compared to less intelligent individuals, thus the result would be in line with the neural efficiency hypotheses. Then we would conclude that neural efficiency reflects a kind of adaption to requirements of the corresponding tasks, which depends on both, intelligence and sample-based task difficulty.

## 2 Method

### 2.1 Participants

Out of a pool of 298 participants who completed an intelligence structure test, 60 people (32 women and 28 men, aged between 18 and 50 years) were selected for this study. Participants were selected on their g-factor score and represented individuals with relatively low average intelligence (IQ range 80–100) or relatively high average to superior intelligence (IQ range 110–130). Two people were excluded from the analysis because of movement artifacts and technical acquisition problems. The final sample thus comprised 58 persons, who were divided into lower (*n* = 28) and higher (*n* = 30) intelligence groups on the basis of their g-factor scores. The lower intelligence group had an average IQ of 93 (*SD* = 5.59) whereas the higher intelligence group had an average IQ of 123 (*SD* = 9.25). Thus, the two extreme groups differed significantly in terms of psychometric g (*t*(56) = − 14.96, *p* < .01; *d* = 2.04). All participants gave written informed consent approved by the local ethics committee and received €15 for their participation in the fMRI test session.

### 2.2 Measures

#### 2.2.1 Intelligence screening

Participants' general intelligence was assessed by means of the intelligence-structure-battery (INSBAT; Arendasy, Hornke, et al., 2008). The intelligence structure battery is a computerized adaptive intelligence test battery based on the Cattell–Horn–Carroll model (cf. McGrew, 2009), which is commonly used in German-speaking countries. In addition to psychometric g, the test battery assesses fluid intelligence (G_f_), crystallized intelligence (G_c_), quantitative knowledge (G_q_), visual processing (G_v_), short-term memory (G_stm_) and long-term memory (G_ltm_). Each of these second-order stratum factors can be measured by means of two or more subtests constructed by means of different approaches to automatic item generation (for an overview: Arendasy and Sommer, 2012a, Arendasy and Sommer, 2013, Irvine and Kyllonen, 2002). All subtests were calibrated by means of the 1PL Rasch model and exhibited good construct and criterion validities (for an overview: Arendasy, Hornke, et al., 2008). In order to obtain a screening measure of psychometric g the following four subtests were completed: figural-inductive reasoning (FID), arithmetic flexibility (NF), verbal short-term memory (VEK) and word meaning (WB). The subtests were selected to cover a broad range of stratum two factors to avoid construct-underrepresentation in estimating psychometric g (cf. Major, Johnson, & Bouchard, 2011). Furthermore, using a more comprehensive estimate of psychometric g that does not include the experimental task itself also bypasses possible problems of retest effects that have been observed for fluid intelligence test (e.g., Arendasy & Sommer, 2013). All subtests were presented as computerized adaptive tests (CAT) with a target reliability corresponding to *α* = .70. Factor loadings obtained with a representative Austrian norm sample were used to estimate the g-factor score based on the subtest results. The factor scores were further converted into IQ scores using the Austrian norm sample.

#### 2.2.2 Number series task

In the fMRI scanner, participants completed number series tasks taken from the subtest numerical-inductive reasoning (NID: Arendasy & Sommer, 2012a) of the intelligence structure battery (INSBAT: Arendasy, Hornke, et al., 2008). The task of the participants was to discover the rules, which govern the number series item and to complete the number series by selecting the number, that completes the series (e.g., 4 8 16 32 64 128 ? correct response: 256). The total item pool of this computerized adaptive test comprised 120 number series, which had been calibrated by means of the 1PL Rasch model (Rasch, 1980). Previous studies already demonstrated that these items measure fluid intelligence and exhibit a g-factor saturation comparable to commonly used figural matrices tests (e.g., Arendasy, Hergovich and Sommer, 2008, Arendasy and Sommer, 2012b). Furthermore, item design features linked to cognitive component processes involved in solving number series accounted for 88% of the differences in the 1PL item parameters. Thus, there is evidence on the construct validity of the number series used in the present study. In the present study a subset of items was taken from the entire item pool. We chose 10 easy items (e.g., 25 28 27 30 29 32 ? correct response: 31; 1PL item difficulty parameter: mean σ = − 1.22; *SD* = 0.38), 10 medium hard items (e.g., 16 10 15 30 24 29 ? correct response: 58; 1PL item difficulty parameter: mean σ = 0.18, *SD* = 0.12) and 10 difficult items (e.g., 32 34 16 38 8 42 ? correct response: 4; 1PL item difficulty parameter: mean σ = 1.57, *SD* = 0.44). The easy and medium hard items were selected so that the lower intelligence group would have a mean person-specific solution probability of .80 and .50, respectively. The medium and hard items were selected to ensure that the higher intelligent participants would have mean person-specific solution probabilities of .80 and .50, respectively. The mean solution probabilities were calculated by estimating participants' NID person parameter on the basis of the g-factor scores using the g-factor loadings of each of the five INSBAT subtests used in the present study.

A control condition was implemented by using 10 number series filled with zeros (e.g., 0 0 0 0 0 0). The zeros were replaced with a number between one and five within 15 until 25 s (e.g., 2 2 2 2 2 2). Participants were asked to respond as quickly as possible and indicate by button press which number was presented.

#### 2.2.3 Experimental design

Participants completed a total of 20 number series tasks in the fMRI scanner. While higher intelligent participants worked on number series of medium and high difficulty, lower intelligent participants completed easy and medium difficult number series. In this manner all participants completed number series with expected mean person specific solution probability of .80 and .50, respectively. Thus, a 2 × 2 design with the between-subject factor IQ GROUP and the within-subject factor person-specific TASK DIFFICULTY could be used to evaluate, whether differences in task performance and brain activation between lower and higher intelligent participants are attributable to group differences in person-specific solution probabilities. In addition, we were able to compare the task performance and brain activation of lower and higher intelligent participants when working on identical item set, since both groups worked on items of medium difficulty (i.e. same sample-based task difficulty; cf. Table 1).

**Table 1:** Design of the study (cells list expected solution probabilities for the NID task performed during fMRI scanning).

		Solution rate		
		Easy	Medium	Hard
Intelligence group	Lower IQ	80%	50%	
	Higher IQ		80%	50%

#### 2.2.4 fMRI acquisition

A 3.0 T Siemens Magnetom Skyra syngo Scanner (Siemens Medical Systems, Erlangen, Germany) with a 32 channel head coil was used. To minimize head movements, subjects' heads were stabilized with foam cushions. Functional images were obtained in 36 slices, in an inter-leaved order. T2*-weighted functional images were obtained with a single shot gradient echo planar imaging (EPI) sequence sensitive to blood oxygen level-dependent (BOLD) contrast (repetition time [TR] 3000 ms, echo time [TE] 30 ms, voxel size 3.5 × 3.5 × 3.5 mm, matrix size 64 × 64, field of view: 192, flip angle [FA] 90°).

**Fig. 1:**
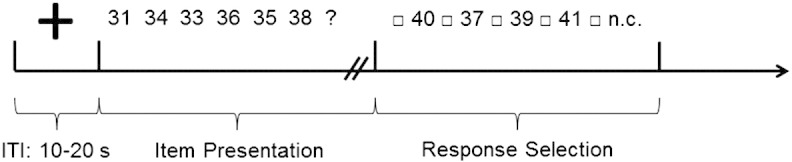
Schematic time course for the number series task. After an inter-trial interval (10–20 s), participants worked on the number series task in a self-paced manner (timeout 60 s). When participants knew the solution they had to press a key and then could select one of the presented numbers. n.c. = no response option correct.

Stimulus presentation was accomplished with an LCD projector, visible for the participant through a mirror mounted above the head coil. The paradigm was presented with the software package Presentation (Neurobehavioral Systems, Albany, CA). In Fig. 1a, schematic display of one trial is depicted. The presentation of the fixation cross (10–20 s) marked the beginning of each trial. After this, the stimulus presentation started (max. 60 s) and the participants had to respond as fast and accurately as possible. If participants knew the correct solution within 60 s, they could respond by key press, effecting that the number series was replaced by five response options. Otherwise the five response options appeared after 60 s. Four numeric response options and the response “none is correct” (to reduce guessing) were presented. For responding, a response box was placed in the participants' right hand. A randomly selected response was highlighted in the beginning. Participants could then move left or right between response options using the left or right button. To confirm the selected response the middle button had to be pressed. Each response was followed by a randomly jittered inter-trial interval of 10–20 s.

#### 2.2.5 fMRI-analyses

Functional MRI scans were analyzed using the Software SPM8 (Wellcome Department of Imaging Neuroscience, London, UK). For each person preprocessing was performed, including motion correction, slice acquisition time correction and spatial normalization into the standard MNI space (Montreal Neurological Institute). Finally, the data were smoothed in the spatial domain using a Gaussian kernel of 10.5 mm FWHM. Due to the self-paced design, the length of the fMRI sequence varied. On average 472 volumes (*SD* = 98) were acquired. A general linear model (GLM) was estimated for each participant, using the conditions ‘FIXATION’ (i.e., inter-trial fixation period), ‘CONTROL’ (i.e., control trials requiring no reasoning), ‘EASY’ (person-specific easy number series items; A vs. B), and ‘DIFFICULT’ (person-specific difficult number series items; B vs. C). For person-specific easy and difficult trials, a constant period representing the first 15 s of each trial was modeled in order to control for activation effects due to variation in response times within and between participants (82% of trials had a response time of 15 s or more, shorter trials were excluded from the analysis). The remaining task time was modeled by a separate regressor of no interest. Finally, motion parameters were included in the model to account for uncorrected motion effects.

For the analysis of task-related activation, a factorial design was implemented. Task-related activation was defined by the contrast EASY and DIFFICULT vs. CONTROL. The analysis for the entire group was performed by computing linear *t*-contrasts for each subject individually which were then entered into the second level analysis, employing a significance level of *p* < .05 (corrected for family wise error [FWE]). Potential intelligence effects on brain activation when working on person-specific easy, person-specific difficult and sample-based equally difficult tasks were analyzed with three two-sample *t*-tests using IQ GROUP as between subjects factor. Linear *t*-contrasts (EASY vs. CONTROL, and DIFFICULT vs. CONTROL) were computed for each subject individually which were then entered into the second level analysis. Results are only presented if they were significant on cluster level (*p* < .001, uncorrected) and exceeded a minimum cluster size of 50 for intelligence differences. The clusters showing significant intelligence group differences were used for the construction of functionally defined ROIs. We then computed contrast estimates within ROIs to determine the magnitude and direction of significant effects.

## 3 Results

### 3.1 Behavioral results

In order to examine possible group differences between more and less intelligent individuals with respect to task performance (solution rate and response time) when completing person-specific easy and difficult number series, a two-way ANOVA with IQ GROUP (lower vs. higher) as between-subjects variable and TASK DIFFICULTY (person-specific easy vs. person-specific difficult) as within-subjects variable was computed. For the analysis of solution rates, the main effect IQ GROUP remained insignificant (*F*(1,56) = 0.13, *p* = .72). As shown in Table 2, a significant main effect TASK DIFFICULTY was observed (*F*(1,56) = 29.38, *p* = .00; *partial* η^2^ = .34). Participants solved more person-specific easy number series correct than person-specific difficult number series. The two-way interaction IQ GROUP ∗ TASK DIFFICULTY was also non-significant (*F*(1,56) = 1.87, *p* = .18).

For the analysis of response times (for correct trials), neither a significant main effect IQ GROUP (*F*(1,56) = 0.14, *p* = .71) nor a significant two-way interaction IQ GROUP ∗ TASK DIFFICULTY (*F*(1,56) = 0.41, *p* = .53) was found. This implies that, although a self-paced design was employed, intelligence groups did not differ in the average time-on-task, which is most likely due to the adaption of task-difficulty to the ability level. The main effect TASK DIFFICULTY was significant, indicating that it took longer to solve difficult number series than easy number series (*F*(1,56) = 29.49, *p* = .00, *partial* η^2^ = .35; Table 2). We then analyzed possible group differences between more and less intelligent individuals with respect to task performance during performing equally difficult number series. As expected, higher intelligent individuals showed a higher solution rate (*t*(56) = − 3.35, *p* < .01, *d* = 0.90) and lower response times (*t*(56) = 2.37, *p* = .02, *d* = 0.63) than lower intelligent individuals when working on identical tasks. To sum up, intelligence group differences in task performance were only found for the subset of tasks showing the same sample-based difficulty, but not when comparing tasks of similar person-specific difficulty.

**Table 2:** Solution rates and response times in the number series task for higher and lower intelligent individuals.

		Solution rate (%)			Response time (s)		
		Easy	Medium	Hard	Easy	Medium	Hard
Intelligence group	Lower IQ	61.79	47.86		24.15	31.45	
	Higher IQ		68.33	45		25.72	31.48

### 3.2 Task-related brain activation

**Fig. 2:**
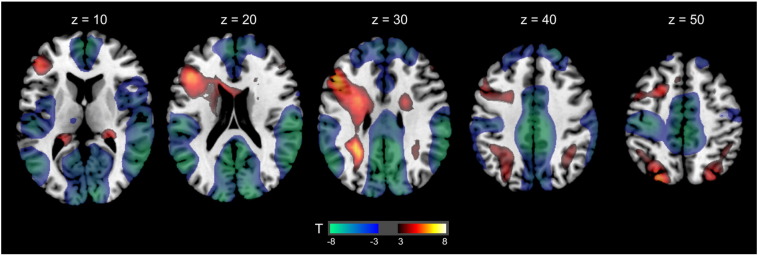
Brain areas activated during number series task (EASY and DIFFICULT vs. CONTROL). The activation period was the first 15 s after the stimulus appeared. Red means relative higher activation during the number series compared to the control task and blue means relative lower activation during the number series compared to the control task. The figure presents 5 axial slices in neurological convention from z = 10 to 50, MNI space.

The contrast EASY and DIFFICULT > CONTROL revealed eight significant clusters (see Table 3). Performance of the number series task was related to brain activation in the left inferior and bilateral regions of the middle frontal gyrus. Activation in the parietal cortex emerged in the superior parietal lobule, bilaterally. Finally, significant activation clusters were found in the cerebellum in the left hemisphere and in the inferior occipital lobule in the right hemisphere. The reverse contrast (CONTROL > EASY and DIFFICULT) returned nine significant clusters in the right frontal, bilateral temporal and parietal cortex (for more detail see Table 3), indicating that these brain regions were more strongly activated in the control task as compared to the reasoning task. Brain areas active during number series task are depicted in Fig. 2. The same clusters were significant when only tasks with same sample-based task difficulty were considered in the analysis.

**Table 3:** Whole brain analysis of brain areas activated during number series tasks.

Brain area	MNI peak coordinate					
	x	y	z	k	*T*	*p*corr
*EASY and DIFFICULT > CONTROL*						
Inferior frontal L (BA 46)	− 50	25	27	242	6.73	.00
Parietal L	− 29	− 49	31	36	6.71	.00
Inferior occipital R (BA 18)	31	− 98	− 5	11	6.12	.00
Superior parietal L (BA 7)	− 22	− 81	52	45	5.88	.00
Superior parietal R (BA 7)	20	− 70	62	27	5.73	.00
Middle frontal L (BA 6)	− 29	11	62	29	5.67	.00
Middle frontal R (BA 6)	34	11	62	6	5.34	.01
Cerebellum L	− 1	− 81	− 26	6	5.33	.01

*CONTROL > EASY and DIFFICULT*						
Precuneus L (BA 31)	− 5	− 60	27	3290	12.11	.00
Inferior parietal lobe R (BA 40)	66	− 28	24	1352	10.27	.00
Angular L (BA 40)	− 54	− 60	24	1076	10.19	.00
Temporal middle L (BA 21)	− 64	− 14	− 22	420	9.07	.00
Medial frontal R (BA 10)	3	56	− 5	763	9.00	.00
Inferior orbital frontal R (BA 38)	31	25	− 26	6	5.49	.01
Superior medial frontal R (BA 6)	13	32	62	4	5.38	.01
Thalamus L	− 12	− 25	− 1	14	5.37	.01
Superior temporal R (BA 38)	34	21	− 33	3	5.3	.01

*Note*. L = left hemisphere, R = right hemisphere, BA = Brodmann area.

### 3.3 Intelligence effects on tasks with the same person-specific task difficulty

In order to examine possible group differences between the lower and higher intelligence groups with respect to brain activation when working on tasks with the same person-specific task difficulty, two *t*-contrasts with IQ GROUP as between-subjects variable were computed, for person-specific easy and difficult items, respectively.

No significant intelligence group differences emerged — neither for easy nor for difficult tasks. This implies that the two intelligence groups did not differ in their brain activation when compared at a person-specific equal level of task difficulty.

### 3.4 Intelligence effects on tasks with the same sample-based task difficulty

**Fig. 3:**
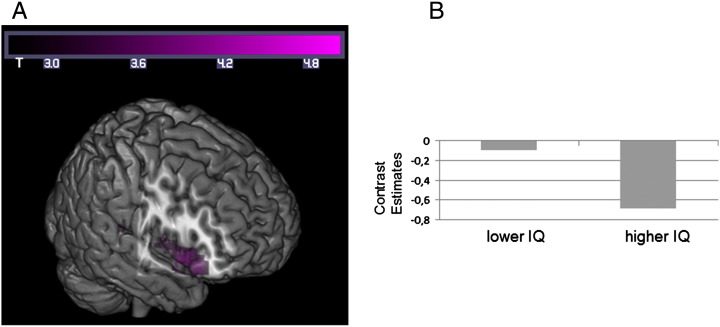
(A) *T*-contrast between intelligence groups when working on tasks with same sample-based task difficulty with a significant cluster in the right insula. (B) Mean contrast estimates of number series task (against control) for higher and lower intelligent individuals when working on tasks with the same sample-based difficulty.

We then analyzed intelligence group effects on brain activation during reasoning tasks with the same sample-based task difficulty on brain activation of less intelligent versus brighter individuals. The *T*-contrast revealed that the right insula is more strongly activated in the lower intelligence group as compared to the higher intelligence group when working on tasks with same sample-based difficulty. In the reverse contrast (higher > lower intelligence) no significant activation cluster were observed. In a second step, individual contrast estimates were computed for the ROI defined by the significant group. The contrast estimates showed that working on the number series compared to the control task resulted in lower relative activation of the right insula in both intelligence groups, but this relative deactivation was significantly stronger in the higher intelligence group (*t*[56] = 3.20, *p* ≤ .01; see Fig. 3).

Moreover, we analyzed intelligence group differences separately for males and females in a SPM whole brain analyses, showing no significant effect by sex.

## 4 Discussion

This study aimed at further examining the effect of person-specific and sample-based differences in task difficulty on the phenomenon of neural efficiency. Our hypothesis was based on research demonstrating that neural efficiency was often observed for easy to moderately difficult tasks but not for hard tasks (Neubauer & Fink, 2009). More specifically, it was tested whether these findings might be attributed to the task demands such as person-specific task difficulty.

Behaviorally, as expected individuals working on tasks with the same person-specific task difficulty performed equally well in the number series task, whereas a classical comparison between brighter and less intelligent individuals working on tasks with the same sample-based task difficulty resulted in lower task performance of less intelligent individuals compared to brighter ones. Furthermore, individual differences in brain activation were only found when working on a task of the same sample-based task difficulty only but not when task difficulty was adapted to the ability of the participants. When confronted with the same sample-based difficulty brighter individuals showed lower activation in the insula than less intelligent individuals. This suggests that neural efficiency reflects some kind of compensatory effort, presumably due to the fact that less bright individuals perceive the same tasks as more difficult. These findings shall now be discussed in more detail.

### 4.1 Behavioral results

Intelligence group differences in task performance were only found for tasks showing the same sample-based task difficulty, but not when using tasks tailored to the individuals' ability. These results reflect the general finding that less intelligent individuals require more time to solve tasks and make more mistakes when working on tasks with the same sample-based task difficulty (e.g., Neubauer et al., 2004, Neubauer et al., 1997, Neubauer et al., 2002). However, when comparing performance while working on tasks with the same person-specific level of task difficulty brighter individuals and less intelligent individuals did not differ in their solution rates and reaction times neither when working on person-specific easy nor when working on person-specific difficult tasks. This result replicates previous findings reported by Larson et al. (1995). As in our study, Larson and colleges used items that were tailored to the participants' own ability levels, thereby standardizing task difficulty.

Thus, it can be assumed that the task demands were the same for IQ groups in the same person-specific task difficulty condition. However, task demands were lower for brighter individuals when working on tasks with the same sample-based task difficulty. These results indicate that the operationalization of the experimental task difficulty conditions was successful.

## 5 fMRI results

As we were specifically interested in whether intelligence differences in neural efficiency can be traced back to the relative task difficulty depending on the individual level of intellectual ability, it is of particular interest to highlight our results on how intelligence groups differ in brain activation when working on tasks with comparable person-specific and sample-based difficulty. When working on person-specific same difficult tasks brighter and less intelligent individuals did not show any activation differences. As we designed the experiment to ensure that task demands were equivalent for the two groups, it can be assumed that invested mental effort (quantified by brain activity) is the same for lower versus higher intelligent individuals when working on tasks showing the same person-specific difficulty.

Intelligence-dependent activation differences supporting neural efficiency were only observed when participants worked on tasks with the same sample-based task difficulty. Specifically, analyses indicated that brighter individuals showed lower relative activity in the right insula than less intelligent individuals.

The insula, together with a fronto-parietal network has been repeatedly related to intelligence (cf. Jung & Haier, 2007). These are brain regions that are assumed to constitute an important network involved in complex information processing. The insula is involved in high-level cognitive control and attentional processes (Menon & Uddin, 2010), such as engaging task-relevant cognitive processes while disengaging task-irrelevant systems (Sridharan, Levitin, & Menon, 2008). The insula is also involved in the bottom-up detection of salient events, the switching between large-scale networks (default-mode network and central-executive network), autonomic reactivity to salient stimuli and the access to the motor system (Menon & Uddin, 2010). The insula as part of the salience network is important for monitoring the salience of external inputs and internal brain events. If relevant stimuli from the vast and continuous stream of sensory stimuli are identified, then the insula is thought to dynamically switch between default-mode network and central-executive network (Bressler & Menon, 2010). The right insula plays a crucial role in activating the central-executive network (mediating attentional, working memory and higher order cognitive processes) and deactivating the default-mode network (Sridharan et al., 2008).

How could the insula finding be related to intelligence and efficiency? It can be assumed that the employed reasoning task strongly taxes the central-executive network for identifying and testing rules underlying the number series, but does not require much sensory salience detection. This is quite different for the control task, which required reacting as quickly as possible to a change of the stimulus. Accordingly, a stronger involvement of the insula (as part of the salience network) in the control task as compared to the reasoning task could be considered as an efficient adaptation of brain activity. This adaptive response is exactly what was found for higher intelligent individuals but only to a significant lower degree in lower intelligent individuals due to a higher person-specific challenge. It hence could be concluded that higher intelligent brains are more efficient in terms of more selectively activating task-relevant brain regions (Lipp et al., 2012). This interpretation is in line with recent findings suggesting that intelligence is related to stronger brain activation in task-positive networks and lower brain activation in task-negative networks (Basten, Stelzel, & Fiebach, 2013). Finally, the findings add to the evidence that complex reasoning can be understood in terms of adaptive activation of large-scale brain networks (cf., Bressler & Menon, 2010). In this context, the results may help caution that subject IQ can affect brain activation, and that IQ hence can be an important factor to consider in cognitive neuroscience research.

Interestingly, our findings are not in line with previous findings by Larson et al. (1995) who found increased activation in brighter individuals and decreased activation in average-intelligent individuals when working on person-specific hard tasks. The discrepant findings could in part be the result of the different imaging methods. While Larson et al. (1995) used PET, this study used an event-related approach with fMRI. Furthermore, the task used by Larson was a backwards digit span task commonly used to assess working memory whereas number series were used in the current study. The different demands underlying backwards digit span tasks and number series could also be a possible explanation for the conflicting results. Finally, the Larson study used somewhat easier item-difficulty levels (Larson et al.: 90% and 75%; this study: 80% and 50%) thus examining brain activation related to a lower degree of cognitive challenge.

As a potential limitation of this study, it should be noted that significant group differences were obtained with a rather liberal threshold criterion (*p* < .001 uncorrected double thresholded with a minimum cluster size of 50 voxel), but would not be significant when applying a conservative threshold such as FWE correction. In part a more sensitive criterion could be justified by the fact that individual differences' analyses usually have lower power than within-subject task contrasts.

## 6 Conclusion

The results provide evidence that neural efficiency is a function of both intelligence and task demands. Results indicate that the neural efficiency hypothesis needs to be refined. According to the refined definition, neural efficiency describes the phenomenon that more intelligent individuals show lower brain activity than less intelligent ones only when working on cognitive tasks with a comparable sample-based difficulty. We hypothesize that this reflects a more efficient adaption of brain activation due to lower person-specific challenge. However, when comparable person-specific challenge is established lower versus higher IQ brains show similar brain activity levels. These results suggest that the neural efficiency phenomenon may actually be explained by the adaption of brain activation to the person-specific task demands.
